# Factors Associated With Multidimensional Sleep Health in Japanese Adults: An Age-Stratified Analysis

**DOI:** 10.1093/geroni/igaf122.770

**Published:** 2025-12-31

**Authors:** Ryuji Furihata, Yukiko Tateyama, Tomonari Shimamoto, Nishioka Norihiro, Takahashi Yoshimitsu, Hiroshi Okada, Takeo Nakayama, Taku Iwami

**Affiliations:** Kyoto University, Kyoto, Kyoto, Japan; Kyoto University, Kyoto, Kyoto, Japan; Kyoto University, Kyoto, Kyoto, Japan; Kyoto University, Kyoto, Kyoto, Japan; Kyoto University, Kyoto, Kyoto, Japan; Wakayama Medical University, Wakayama, Wakayama, Japan; Kyoto University, Kyoto, Kyoto, Japan; Kyoto University, Kyoto, Kyoto, Japan

## Abstract

**Objective:**

To investigate factors associated with multidimensional sleep health among community-dwelling adults and to assess variations by age group.

**Methods:**

A self-administered survey was conducted from December 2022 to February 2023 among Kyoto City residents aged 40 years or older. Multidimensional sleep health was measured using the RU_SATED questionnaire, which evaluates regularity, satisfaction, alertness, timing, efficiency, and duration. A score of 10 or higher defined good sleep health. Multivariable logistic regression was used to investigate associated factors. The study was approved by the Kyoto University Ethics Committee.

**Results:**

Data from 661 respondents (57.9% female, mean age 64.9 years) were analyzed. Good sleep health was observed in 45.8% of participants, including 44.5% of those younger than 75 years and 49.7% of those 75 years or older. Age group and alcohol drinking (OR = 1.64) were positively associated with good sleep health. In participants younger than 75 years, exercise for health (OR = 1.60) and alcohol drinking (OR = 1.83) were positively associated with good sleep health. In those 75 years or older, obesity (OR = 0.40) was negatively associated with good sleep health.

**Conclusion:**

Factors associated with good sleep health differ by age group. These findings may provide important evidence for community-based health promotion focused on multidimensional sleep health.

